# Coexistence of two different mutations in codon 12 of the Kras gene in colorectal cancer: Report of a case supporting the concept of tumoral heterogeneity

**DOI:** 10.3892/ol.2013.1255

**Published:** 2013-03-14

**Authors:** GIUSEPPINA IMPROTA, ANGELA ZUPA, LUCIANA POSSIDENTE, ALFREDO TARTARONE, PIERNICOLA PEDICINI, ANTONIO NAPPI, SERGIO MOLINARI, FILIPPO FRAGGETTA, GIULIA VITA

**Affiliations:** 1 Laboratory of Clinical Research and Molecular Diagnostics, IRCCS-CROB Hospital, Rionero In Vulture, Potenza;; 2 Departments of Medical Oncology, IRCCS-CROB Hospital, Rionero In Vulture, Potenza;; 3 Nuclear Medicine and Radiotherapy, IRCCS-CROB Hospital, Rionero In Vulture, Potenza;; 4 Healthcare Management, IRCCS-CROB Hospital, Rionero In Vulture, Potenza;; 5 Department of Pathology, Cannizzaro Hospital, Catania;; 6 Department of Pathology, IRCCS-CROB Hospital, Rionero In Vulture, Potenza, Italy

**Keywords:** KRAS, colorectal cancer, multiple mutations, tumoral heterogeneity

## Abstract

Evaluation of the mutational status of KRAS is a crucial step for the correct therapeutic approach in treating advanced colorectal cancer as the identification of wild-type KRAS tumors leads to more specific and less toxic treatments for patients. Although several studies have highlighted the differences between primary and metastatic tumors, the possibility of two or more mutations in the same codon has seldom been reported. The present study reports an additional case of an advanced adenocarcinoma of the colon showing two somatic mutations (p.G12D and p.G12V) in the same codon (codon 12) of exon 2 of the KRAS gene, thus supporting the possibility of two differing clonal origins of the tumor. Although the clinical significance of multiple mutations remains unknown at present, based on the limited data available in the literature, this rare event appears to be associated with a more aggressive disease, as in the present case. This case report demonstrates the existence of intratumoral heterogeneity and the coexistence of distinct clones within a tumor that may have profound clinical implications for disease progression and therapeutic responses.

## Introduction

Evaluation of the KRAS mutational status is a crucial step for the correct therapeutic approach in advanced colorectal cancer. According to well-established criteria, a molecular analysis of exon 2 (codons 12 and 13) is routinely performed in formalin-fixed, paraffin-embedded (FFPE) tissue and the identification of a wild-type (WT) KRAS tumor may lead to a more tumor-specific and less toxic treatment for the patient.

Numerous studies have demonstrated significant intratumoral heterogeneity, with spatially separated heterogeneous somatic mutations and chromosomal imbalances (1). With regard to colorectal cancer, several studies have highlighted the differences in the KRAS mutational status between primary and metastatic tumors within lymph nodes and visceral metastases or even different portions of the primary lesion (2), thus supporting the overall current theory of neoplastic heterogeneity (1,3). However, the possibility of two or more mutations in the same codon of the KRAS gene has seldom been reported in colorectal cancer and the real clinical impact of multiple mutations on patient prognosis has not yet been well studied and clarified (4–7).

The present study reports an additional case of the coexistence of two somatic mutations (p.G12D and p.G12V) in the same codon (codon 12) of exon 2 of the KRAS gene in a female patient affected by an advanced adenocarcinoma of the rectum. This supports the possibility for two clonal origins of the tumor along with concomitantly different mutations at the same genetic level.

## Case report

In March 2012, a 70-year-old female patient was admitted to the IRCCS-CROB Hospital (Rionero In Vulture, Italy) due to tenesmus and blood in the stool. The subsequent colonoscopy examination showed a hyperemic flat lesion of the rectal mucosae with rectal canal stenosis. A rectal biopsy supported the diagnosis of poorly differentiated adenocarcinoma. Subsequent to a careful clinical evaluation, including a total body computed tomography (CT) scan, the patient was diagnosed as having locally advanced rectal cancer cT3N1M0, stage IIIb.

Between April and June 2012, the patient received neoadjuvant chemotherapy with 1,650 mg/m^2^ capecitabine orally twice daily, plus concomitant radiotherapy (45 Gy in 5 weeks), followed 6 weeks later by surgery. In August 2012, the patient underwent an anterior resection for rectal cancer. The histological examination revealed a ypT3 adenocarcinoma, of tumor regression grading (TRG) 3 with metastases in 1 out of 10 resected lymph nodes (ypT3pN1Mx TRG3).

In October 2012, prior to starting the planned post-operative chemotherapy, a CT scan revealed multiple liver metastases, although the carcinoembryonic antigen (CEA) and carbohydrate antigen (CA) 19.9 values were normal. The patient began chemotherapy with 7.5 mg/kg bevacizumab daily and the XELOX regimen (130 mg/m^2^ oxaliplatin daily + 2,000 mg/m^2^ capecitabine on days 1–14). This treatment is ongoing.

### Materials and methods

Prior to the mutation analysis, the patient provided informed written consent. A tissue sample from the primary tumor was obtained from the archives of the IRCCS-CROB Hospital. A section of this specimen, stained with hematoxylin and eosin, was observed by a pathologist to evaluate the percentage of cancer cells prior to performing a manual dissection of the tumor area. DNA extraction was performed using a QIAamp^®^ DNA Mini kit (Qiagen, Hilden, Germany), and exon 2 (codons 12 and 13) of the KRAS gene was amplified by polymerase chain reaction (PCR) using the following designed primers: forward, 5′-GGTGGAGTATTTGATAGTGTAT-3′ and reverse, 5′-AGAATGGTCCTGCACCAGTAA-3′. PCR was performed in a final volume of 25 *μ*l under the following conditions: 1X buffer, 3 mmol/l magnesium chloride, 200 *μ*mol/l deoxyribonucleotide phosphates (all from Applied Biosystems, manufactured by Roche, Branchburg, NJ, USA), 12.5 pmol of each primer (Sigma Aldrich, St. Louis, MO, USA), 200 ng/*μ*l DNA and 1.5 U Taq DNA polymerase (Applied Biosystems manufactured by Roche). Following use of an initial melting temperature of 94°C for 2 min, the reaction mixture was subjected to 35 cycles of 94°C for 30 sec, 56°C for 30 sec and 65°C for 30 sec, followed by a final 72°C extension step for 2 min. Prior to sequencing, the PCR products were stained with ethidium bromide and visualized on a UV transilluminator following 2% gel electrophoresis. The PCR products were then sequenced using the BigDye Terminator v.1.1 sequencing kit and an ABI Prism 310 genetic analyzer (both from Applied BioSystems, Foster City, CA, USA) (8).

### Results

**Figure 1:**
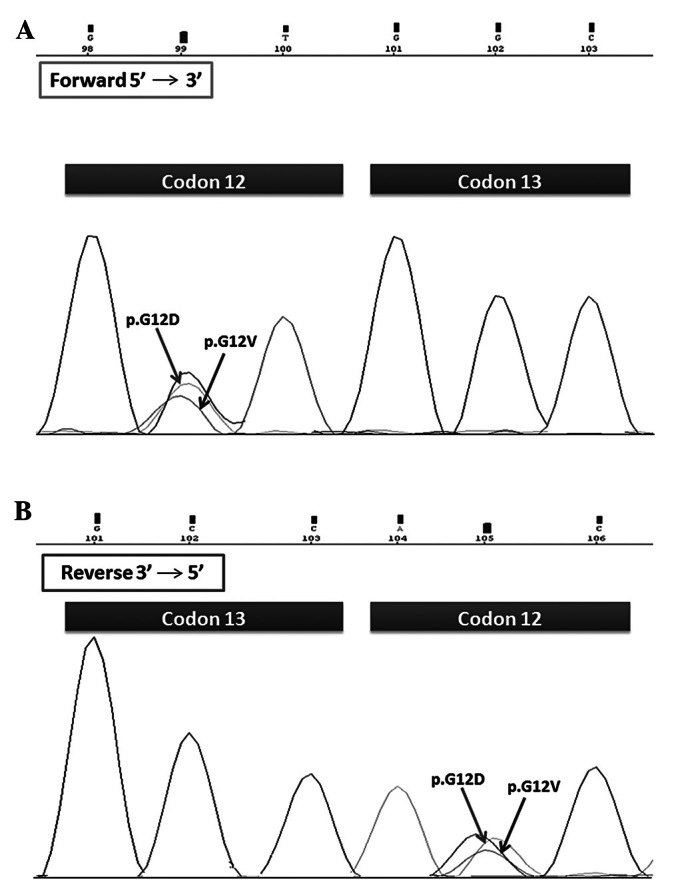
Direct sequencing analysis of codons 12 and 13 of the KRAS gene (exon 2) showing the presence of two mutations, p.G12D and p.G12V, in the same nucleotide position of codon 12, in the (A) forward and (B) reverse directions.

The percentage of sample tumor cells was evaluated by the pathologist and estimated to be ∼60%. The DNA quality was evaluated by spectrophotometer analysis and the A_260_/A_280_ ratio was 1.8. The coexistence of two mutations, p.G12D and p.G12V, in the same codon (codon 12) of the KRAS gene was observed by molecular biologists in two independent PCR products and demonstrated by sense and anti-sense sequence analysis of the fragments (Fig. 1). The DNA amplified sequence of the KRAS gene was compared with the wild-type KRAS sequence.

## Discussion

Colorectal cancer is the third most commonly diagnosed type of cancer and the third leading cause of cancer mortality in males and females. With the development of drugs, including irinotecan and oxaliplatin, and targeted therapies, including cetuximab and bevacizumab therapy, the median survival has increased to >20 months. Several studies have shown that KRAS mutations in primary tumors predict resistance to anti-EGFR antibodies (9–11) and thus, only patients with wild-type KRAS tumors (∼60% of patients) are eligible for anti-EGFR therapy.

Although the results of the KRAS mutational analysis of the primary tumor usually match with the metastases, in a minority of cases (5–10%), the KRAS mutational status is heterogeneous between the primary tumor and metastases (2,12–15). These observations may reflect the increased genetic instability in cells that progressively acquire mutations or the presence of a heterogeneous group of neoplastic cells inside the tumor (16,17). In addition, few studies have observed the coexistence of more than one mutation in the KRAS gene within the same colorectal tumor, correlating this type of alteration with clinical and morphological features (4–7).

The present study reports a case of the coexistence of two mutations, p.G12D (GGT>GAT) and p.G12V (GGT>GTT), in the same codon of the KRAS gene, in the same selected tumor area, thus demonstrating the existence of intratumoral heterogeneity. Based on data in the literature, multiple mutations in the KRAS gene are infrequent, representing 2.1% of mutations in colorectal cancer (18). The majority of co-mutations in the KRAS gene affect only one codon (59%), mainly codon 12, although co-mutations may affect codons 12 and 13 simultaneously (18). The most frequently altered amino acid sequences involved in these co-mutations are GAT (in codon 12) and GAC (in codon 13) (18).

Due to the scarcity of data in the literature, the clinical implications and prognostic significance of multiple KRAS mutations remain unknown, although associations with advanced clinical stage and aggressive clinical course have been reported (4–7,19). The present case was characterized by an aggressive clinical course with the development of early liver metastases despite the administration of neoadjuvant chemo-radiotherapy. Whether this aggressiveness was due to the coexistence of multiple mutations or a specific single mutation is, however, questionable.

It has been shown that not all KRAS mutations have the same prognostic relevance. A meta-analysis demonstrated that the p.G12V mutation at codon 12 in the KRAS gene increases the risk of recurrence or mortality in patients with colorectal cancer (17,20–21), unlike other KRAS mutations that have only a moderate, non-significant effect on overall survival (22). These data are consistent with experimental evidence showing that valine mutations produce proteins with different behavior compared with other mutated KRAS proteins (12). The lower affinity of GTP to p.G12D allows p.G12D to escape from the oncogenic GTP-bound state, whereas GTP that is tightly bound to p.G12V generates a more persistent, potentially oncogenic signal. Furthermore, differences in the effector region of p.G12D and p.G12V may modify interactions with downstream signaling molecules (12).

In the present case the coexistence of these two mutations, p.G12D and p.G12V, may have had an almost different clinical relevance on patient prognosis (20,22). The effect of various KRAS mutations on overall survival may be explained by the fact that the heterogeneity of the various KRAS mutations in colorectal cancer may differ in carcinogenic potential. This may justify the selection of a new clone with a p.G12V mutation and more aggressive behavior, along with the pre-existing p.G12D clone.

Therefore, it should be a great challenge to detect all mutations present in tumors. It has been suggested that a DNA tumoral mix obtained from different tumor areas may increase the detection rate of mutations, including multiple mutations (2,23). This is consistent with the current theory that tumors show significant intratumoral heterogeneity, characterized by separated heterogeneous somatic mutations and chromosomal aberrations (1). Such genetic heterogeneity may also cause heterogeneitiy in terms of radiosensitivity (24), with a strong impact on the choice of the most appropriate treatment option when the disease is treatable with radiotherapy alone or combined with chemotherapy or biological drugs (25,26).

In conclusion, the present study underlines the intratumoral heterogeneity, as supported by the current data (1,2) in which tumors may be polyclonal with a mixture of cell populations harboring varying mutations. The coexistence of distinct clones within a tumor may have profound clinical implications for disease progression and therapeutic responses. In particular, the present case appears to support the hypothesis that the presence of multiple mutations in codon 12 is associated with a more aggressive disease.
